# Microneedle‐based intradermal delivery of stabilized dengue virus

**DOI:** 10.1002/btm2.10127

**Published:** 2019-02-25

**Authors:** Michelle E. Turvey, Divakara S.S.M. Uppu, Abdul Rahim Mohamed Sharif, Katell Bidet, Sylvie Alonso, Eng Eong Ooi, Paula T. Hammond

**Affiliations:** ^1^ Infectious Diseases IRG Singapore‐MIT Alliance for Research and Technology Singapore; ^2^ Department of Microbiology & Immunology, Yong Loo Lin School of Medicine, Immunology Programme Life Sciences Institute, National University of Singapore Singapore; ^3^ Emerging Infectious Diseases Duke‐NUS Graduate Medical School Singapore; ^4^ Koch Institute for Integrative Cancer Research, Massachusetts Institute of Technology Cambridge MA; ^5^ Department of Chemical Engineering Massachusetts Institute of Technology Cambridge MA

**Keywords:** dengue, immunization cold chain, microneedles, vaccine delivery

## Abstract

Current live‐attenuated dengue vaccines require strict cold chain storage. Methods to preserve dengue virus (DENV) viability, which enable vaccines to be transported and administered at ambient temperatures, will be decisive towards the implementation of affordable global vaccination schemes with broad immunization coverage in resource‐limited areas. We have developed a microneedle (MN)‐based vaccine platform for the stabilization and intradermal delivery of live DENV from minimally invasive skin patches. Dengue virus‐stabilized microneedle arrays (VSMN) were fabricated using saccharide‐based formulation of virus and could be stored dry at ambient temperature up to 3 weeks with maintained virus viability. Following intradermal vaccination, VSMN‐delivered DENV was shown to elicit strong neutralizing antibody responses and protection from viral challenge, comparable to that of the conventional liquid vaccine administered subcutaneously. This work supports the potential for MN‐based dengue vaccine technology and the progression towards cold chain‐independence.

Dengue virus can be stabilized using saccharide‐based formulations and coated on microneedle array vaccine patches for storage in dry state with preserved viability at ambient temperature (VSMN; virus‐stabilized microneedle arrays).

## 1 INTRODUCTION

There are two main criteria that denote a successful vaccine—it must elicit life‐long protective immunity against disease from wild‐type microbial infection and it must be accessible by those who live in pathogen endemic areas. Lessening the dependence on cold chain infrastructure reduces vaccine cost and improves the capacity to distribute doses to geographically remote communities.^1^, ^2^, ^3^ The controlled temperature chain (CTC) involves final sections of vaccine transport at ambient temperature through infrastructure‐weak areas and has the potential to reduce vaccination cost by up to 50%.^1^, ^3^, ^4^

Virus vaccines represent a significant challenge in the transition towards CTC implementation. Most virus vaccines are live‐attenuated vaccines (LAVs), which comprise viable virus particles that infect host cells in order to trigger an immune response akin to the natural infection. LAVs induce both humoral and cellular adaptive immunity with robust memory responses; however, despite the immunological advantages, LAVs currently require stringent cold chain for shipment. This cold chain is especially critical for enveloped viruses, such as flaviviruses, that are more unstable than nonenveloped viruses such as poliovirus.

Our efforts are directed towards the development of a vaccine against dengue virus (DENV). DENV infection is the leading cause of mosquito‐borne illness throughout the tropical world, where an estimated 2.5 billion people live at risk of infection each year.^5^, ^6^ Travelers to dengue endemic countries are also at risk of disease and facilitating viral transmission.^6^, ^7^ Since the isolation of DENV in 1943, the development of an effective vaccine that provides balanced, long‐lasting immunity has been challenging. This is partly due to the complexity of dengue epidemiology, population immunity, and the interplay between viral pathogenesis and host immunological mechanisms.^8^, ^9^, ^10^, ^11^, ^12^ Like other successful flavivirus vaccines against yellow fever and Japanese encephalitis, the majority of dengue vaccine candidates either approved for limited license or in Phase III trials are LAVs.^8^, ^13^, ^14^, ^15^ These require shipment in lyophilized form at 4 °C and must be reconstituted immediately before use to avoid loss in potency.^16^, ^17^ Therefore, systems enabling the preservation of DENV viability in dry formulation and at ambient temperatures will be essential for the development of LAVs that can be delivered and administered under CTC conditions.

Flaviviruses, such as DENV, are transmitted by mosquitos following skin penetration by the mosquito fascicle, nature's ideal polymeric microneedle (MN) composed of chitin. The virus infects and replicates within dermal dendritic cells and Langerhans cells, which traffic to lymph nodes and are responsible for processing and presentation of viral antigens during the induction of antiviral immune responses.^18^, ^19^ Intradermal delivery of dengue LAVs would leverage this immune activation in the skin. Hence, another attractive approach for dengue vaccination involves the use of minimally invasive MN‐based skin patches. MNs have been successfully used to deliver a broad range of small molecule drugs, biotherapeutics and vaccines to the skin and mucosal surfaces.^20^ By mimicking the immunological context of natural dengue infection through the skin, native pathways are activated to elicit robust protective immunity.^21^, ^22^, ^23^ Furthermore, MN‐based therapeutics offer additional logistical benefits including low cost of materials, and easy pain‐free administration, which lessens the dependence on trained medical staff and offers the potential for improved compliance.^20^, ^24^, ^25^

We aimed to develop a dengue LAV vaccine platform that combined the advantages of intradermal delivery using MNs with a tailored stabilizing formulation optimized to preserve virus viability. Here we describe the fabrication and efficacy of dengue virus‐stabilized microneedle arrays (VSMNs) formulated to enable virus vaccine storage in the dry state at ambient temperature. VSMNs applied to the skin were shown to induce protective immunity comparable to that of freshly prepared liquid virus. These steps represent important advances towards the development of intradermal dengue vaccines designed for the CTC supply chain.

## 2 RESULTS AND DISCUSSION

In the past decade, numerous MNs‐based platforms have been designed for vaccination against infectious disease,^24^ including solid, coated, dissolvable and hollow architectures. Coated and dissolvable MNs represent dry formulations, which compared to liquid‐based injectable vaccines are easier to store, require lesser space and weight capacity for storage, and have reduced cost of transport. Such designs have been developed for viruses including influenza,^22^, ^26^, ^27^, ^28^, ^29^, ^30^, ^31^, ^32^, ^33^, ^34^, ^35^, ^36^, ^37^ hepatitis B,^38^, ^39^, ^40^, ^41^ hepatitis C,^42^ polio,^43^, ^44^ herpes simplex,^45^, ^46^ human papillomavirus,^47^, ^48^ rotavirus,^49^ measles and rubella,^50^, ^51^, ^52^ rabies,^53^ HIV,^54^, ^55^ Ebola,^56^ chikungunya, and West Nile virus.^57^ However despite extensive advances in MN‐based vaccines, there are limited studies addressing the delivery of LAVs. With the exception of measles and rubella, the aforementioned studies involved the formulation of inactivated virus, virus‐like particles, protein subunit or DNA vaccines, which can be more easily stabilized but forfeits immune exposure to the live infection.

Only measles and rubella have been successfully adapted for live virus vaccination using MN. Studies involved the intradermal delivery of virus from solid coated^50^ or dissolvable MN.^51^, ^52^ With formulation as dissolvable MN vaccine arrays, live measles could be stabilized at 25 °C for months and was demonstrated to induce protective immunity in macaques.^51^, ^52^ Even in the absence of formulation, measles showed considerable innate stability, where viral infectivity was retained for up to 7 days at 25 °C after reconstitution.

By contrast, the most significant hurdle facing MN‐based live DENV vaccines is overcoming the inherent instability of DENV in order to maintain viral potency in ambient conditions. Therefore, we aimed to achieve four goals—(a) to develop formulations to coat DENV on MN arrays for retained infectivity in the dry state at ambient temperature, (b) to demonstrate that formulation‐stabilized DENV could be delivered intradermally from MN to initiate viral infection required for vaccine efficacy, (c) to validate that vaccination with stabilized DENV delivered using MN can induce equivalent immune protection to freshly prepared liquid virus administered subcutaneously, and (d) to optimize formulation for MN vaccine storage for extended periods of time.

### 2.1 DENV formulation for enhanced stability

**Figure 1:**
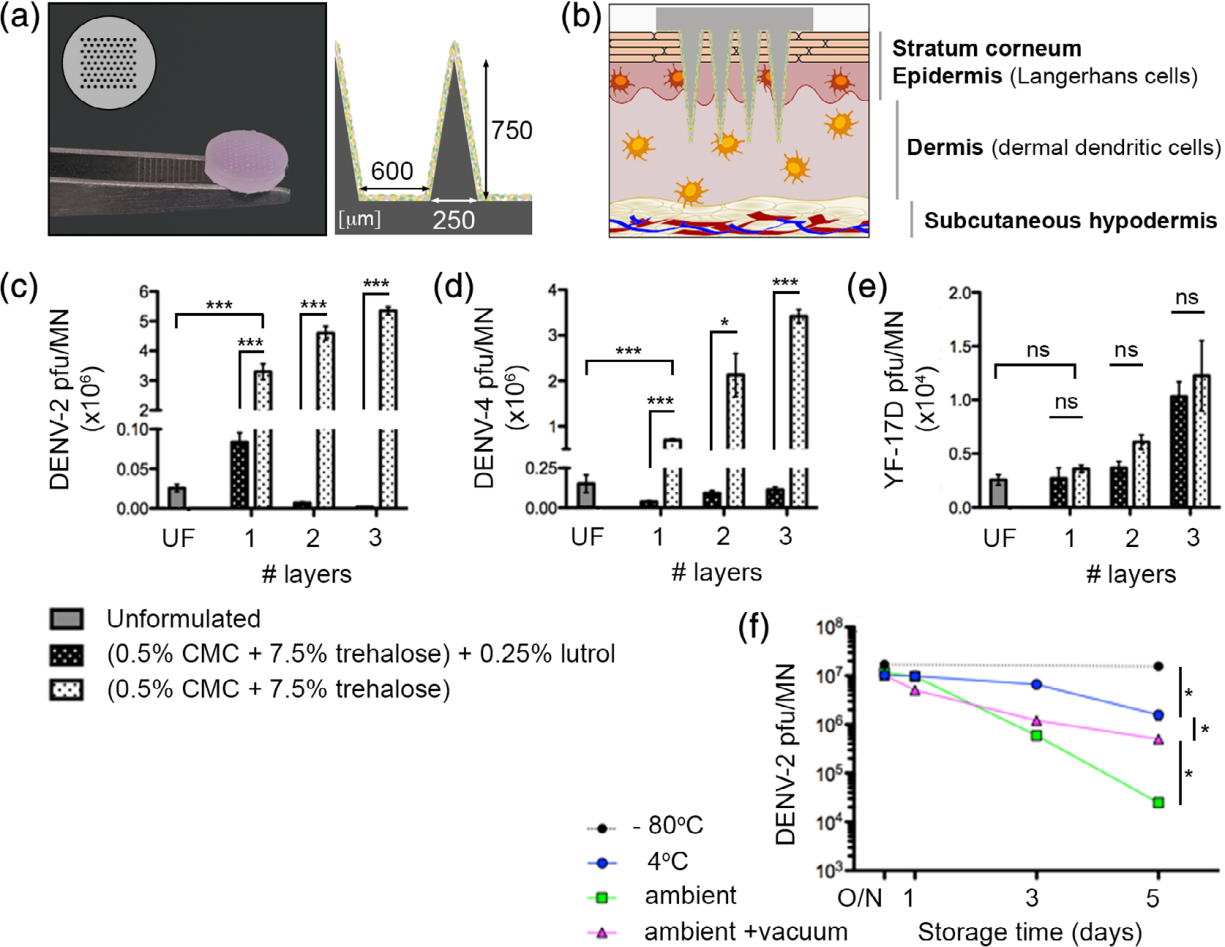
Design and fabrication of virus‐stabilized microneedle arrays (VSMN). Viruses were formulated for improved viability in dry state and short‐term storage at ambient temperature. (a) Representative VSMN and (b) schematic of VSMN intradermal penetration of human skin. Surfactant Lutrol F68 is detrimental to dengue virus (DENV) viability; VSMN fabricated with 1, 2, or 3 layers of (c) DENV‐2 16681 (5 × 10^7^ pfu/layer), (d) DENV‐4 2270 (1.5 × 10^6^ pfu/layer), or (e) YF‐17D (1 × 10^5^ pfu/layer), as unformulated (UF; single layer) or with base formulation (0.5% carboxymethyl cellulose [CMC], 7.5% trehalose) ± 0.25% Lutrol F68. Virus viability determined by plaque assay; *n* = 3 VSMN per condition; *t* test, *p < 0.05; ***p < 0.001; ns, not significant. (f) Stability of DENV‐2 VSMN stored at −80 °C, 4 °C, or ambient temperature (25 ± 2 °C) ± vacuum with desiccant. VSMN fabricated with initial virus input of 8 × 10^7^ pfu/MN. Virus viability determined by plaque assay; *n* = 4 VSMN per condition; Mann–Whitney test of relative values at Day 5, *p < 0.05

Solid poly (l‐lactide) MN array patches were chosen as the base substrate for virus deposition due to their material strength, allowing robust penetration through the outer stratum corneum to underlying skin layers. Patches were fabricated by melt‐molding and comprised arrays with alternating rows of 9 and 8 conical shaped MN projections, each with 250 μm base diameter, 750 μm height and 600 μm pitch (Figure 1a). Similar designs have been used for drug delivery applications.^20^ VSMNs were produced by drop casting saccharide‐formulated virus directly on to the array surface, which was allowed to dry, resulting in a thin layer of virus coating the surface of the needles and the array base. The process of coating and drying could be repeated for multiple layers in order to control the amount of deposited virus. Upon skin application, VSMN enable penetration to the epidermal and dermal layers of human skin, as depicted (Figure 1b). For the generation and maintenance of immunity, the skin is more than a physical barrier. The underlying epidermal and dermal layers compose a rich immune tissue densely populated with resident and recruited immune cells that react rapidly to pathogen insult and potentiate responses in draining lymph nodes. Viral delivery to these skin layers is relevant here for multiple reasons, (a) penetration of up to 1.5 mm depth is considered minimally‐invasive and painless due to lack of innervation, (b) the depth of penetration mimics viral transmission by female *Aedes* mosquitos that have an average fascicle length of 1.8 mm,^58^ and (c) these layers are resident to dermal dendritic cells and Langerhans immune cells.

In agreement with prior MN‐based systems,^30^, ^50^, ^59^, ^60^ the transition from liquid phase to dried state represented a formulation challenge with regards to maintaining vaccine activity. Virus inactivation and aggregation can occur as a result of film dehydration. Based on prior work concerning the stabilization of live measles and inactivated influenza viruses,^27^, ^28^, ^30^, ^50^, ^59^, ^60^ saccharide‐based formulations were optimized in order to retain the viral potency of DENV coated on MN arrays. Two DENV serotypes were assessed, DENV‐2 and DENV‐4, and compared to the clinically approved yellow fever virus LAV strain (YF‐17D). VSMN were dried, kept at ambient temperature for 12 hr, and then the viability of deposited virus was determined by plaque assays of the solubilized formulation. As expected, after drying, unformulated DENV retained minimal viability on MN. In contrast, an optimized formulation of DENV with 0.5% carboxymethyl cellulose (CMC) and 7.5% trehalose during VSMN fabrication resulted in significant preservation of DENV‐2 and DENV‐4 viability (Figure 1c and d) compared to the unformulated control. The importance of CMC for viscosity control during film formation and trehalose as a stabilizer were consistent with prior studies.^30^ Interestingly, of the various excipients tested in this study, the most influential component affecting the viability of DENV was found to be Lutrol F68. Lutrol F68 is a nonionic surfactant that is used during the production of coated MN arrays, where it acts as a wetting and dispersal agent for viscous solutions. Previously it has been used to formulate recombinant adenovirus and modified vaccinia virus Ankara vectors onto solid MN arrays.^61^ Whereas here, even at low concentrations of 0.25% (below critical micellar concentration of 2–3% w/v), Lutrol F68 was shown to completely abrogate the viability of DENV VSMN to levels similar to that of unformulated virus (Figure 1c and d). It could be proposed that the amphiphilic structure of Lutrol F68 led to the destabilization of the viral particle membrane or interacted in a way that prevented virus infection into cells. By contrast, Lutrol F68 did not significantly affect the viability of YF‐17D (Figure 1e), which shares similar membrane architecture to DENV and other related flaviviruses.^62^ YF‐17D also showed more robust viability in the absence of formulation compared to DENV. A mechanistic explanation for stated differences between DENV and YF‐17D viruses was not pursued in the present study, albeit these observations highlight the natural instability of DENV. Collectively, these results indicate that saccharide formulations can be used to retain DENV viability in dry film coatings on MN. In all subsequent experiments, concentrated DENV‐2 with a base formulation of 0.5% CMC and 7.5% trehalose was utilized for VSMN production in a single layer dried film.

Next, the stability of DENV VSMNs was monitored over a 5‐day period with different storage conditions, as measured by DENV‐2 viability. Storage of dry VSMN at −80 °C did not result in loss of infectious dose, whereas storage at 4 °C or ambient temperature was shown to result in incremental decreases in DENV‐2 viability with time (Figure 1f). For ambient temperature conditions, VSMN were stored either “on the bench” or under vacuum containment with desiccant to limit humidity, and as expected, exposure of dry VSMN to humidity negatively affected virus viability. After 5 days storage with limited humidity at ambient temperature, the viability of DENV‐2 VSMN could be successfully stabilized to within 2‐log plaque forming units (pfu)/MN relative to freshly prepared VSMN (Figure 1f).

### 2.2 Intradermal delivery of DENV from VSMN

**Figure 2:**
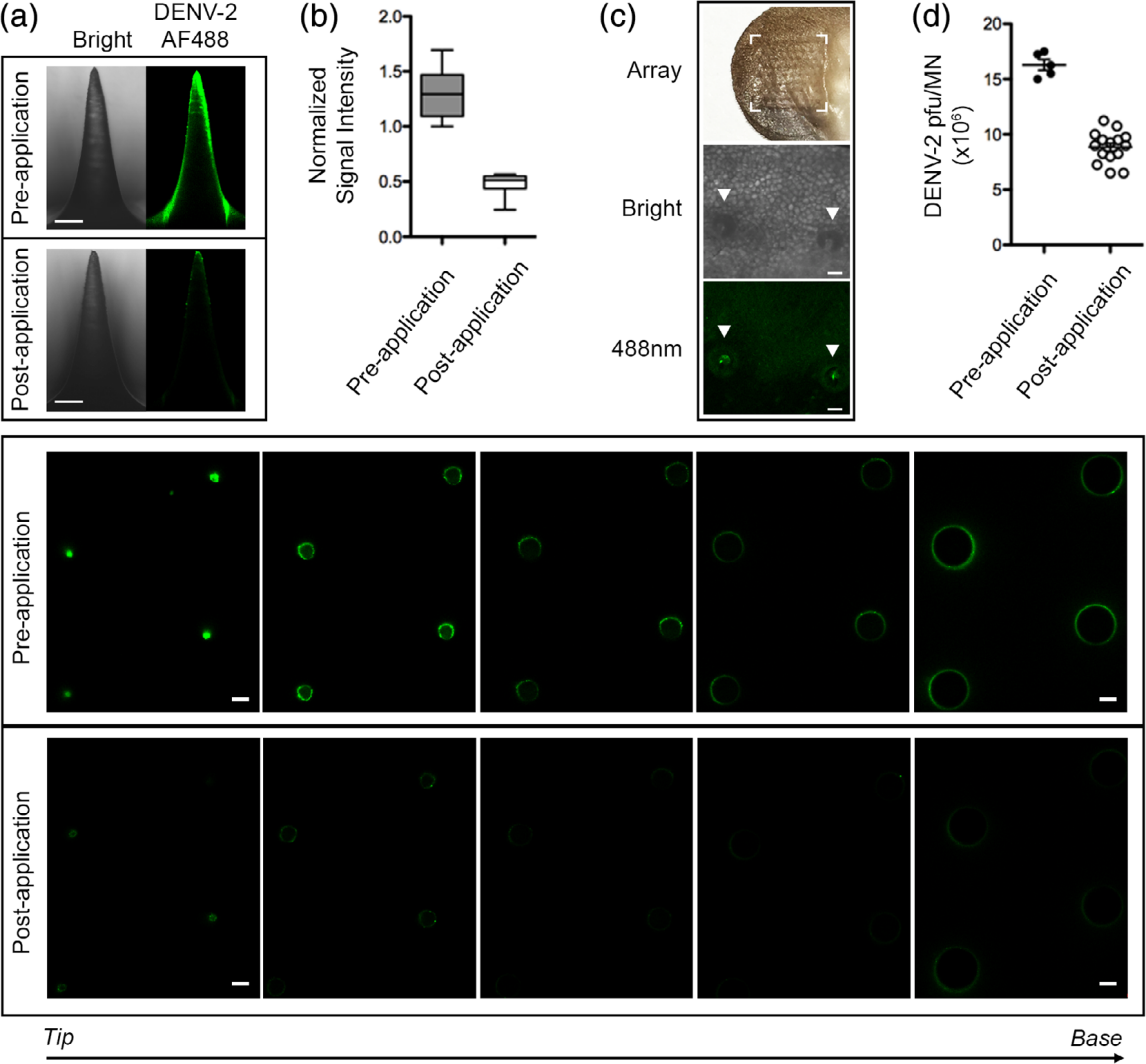
Intradermal delivery from virus‐stabilized microneedle arrays (VSMN). Microneedles (MN) coated with Alexa Fluor 488‐labeled DENV‐2 were applied to mouse ear skin. (a) VSMN imaged before and after 15 min application, as bright field and 488 nm from side view (upper panels), and cross‐sectional images of 4 MN projections captured from tip to base (lower panels); 100 μm z‐intervals, 10× objective, scale bars represent 100 μm. (b) Quantification of normalized signal intensity of MN side view images; data shown as box plot; *n* = 15 projections. (c) Mouse ear skin, imaged in bright field (middle) and at 488 nm (lower) following DENV‐2‐AF488 VSMN application, representative images shown, puncture sites are highlighted with arrows; 10× objective. (d) Quantification of viable virus solubilized from DENV‐2 VSMN; freshly prepared VSMN pre‐application (*n* = 5) and VSMN following application to ear skin (*n* = 15), as determined by plaque assay

To demonstrate the delivery of dry stabilized virus from VSMN to mouse ear skin, an Alexa Fluor 488‐labeled DENV‐2 was used for visualization. Prior to application, a thin film of virus was observed coating the length of the MN surface, which was transferred to the skin following a 15‐min application, as shown by representative images (Figure 2a) and corresponding quantitative fluorescence intensity analyses of *Z*‐sections (Figure 2b). Image analyses of needle projections penetrating the skin (needle surface area represents ~57% of total coated surface area of the patch) revealed approximately 62% reduction in fluorescent signal following skin penetration. Corresponding punctate penetration sites on the ear skin were visualized (Figure 2c).

As a second method to assess the extent of virus delivery from VSMN, plaque assays were performed to quantify the load of viable virus coated on VSMN prior to and remaining after application. This indicated that approximately 50% of the total coated virus was delivered to the skin (Figure 2d). This included virus transferred from needles that penetrate into the skin, in addition to virus transferred from the array base to the skin surface. It is difficult to determine the precise dose of virus that infects host cells in the skin. However, the replication of delivered virus enables the amplification of antigenic material and inflammatory signals that promote the immune response following vaccination. While a simple coated MN design was favored for this study, future vaccines must permit dose standardization to meet quality control and manufacturing standards. Recent work by ourselves and others have achieved this by focusing antigenic components within the tip of dissolvable MNs.^28^, ^51^, ^63^, ^64^

### 2.3 VSMN mediate viremia and induce antiviral immune responses

After developing a saccharide‐based formulation for retained DENV viability on MN, we moved to investigate whether dry stabilized virus delivered intradermally from VSMN was capable of replication *in vivo*. This is important because the potency of LAV vaccines depend on viral replication and infection to produce an effective immune response against the virus. We used DENV‐2 16681 to characterize and compare the viral replication kinetics and tissue localization following VSMN intradermal administration relative to subcutaneous inoculation. Control virus delivered subcutaneously was freshly prepared and injected by hypodermic needle as a benchmark of gold standard. Wild‐type mice do not support DENV replication,^65^ and there are limited small animal models for dengue research, none of which sufficiently recapitulate all aspects of natural infection. DENV‐2 16681 has been shown to result in reproducible viremia in the AG129 (Types I and II interferon receptor deficient) mouse infection model.^65^ The AG129 model allowed the evaluation and comparison of DENV delivery, infection and corresponding antiviral humoral immune responses induced by the different administration routes.

**Figure 3:**
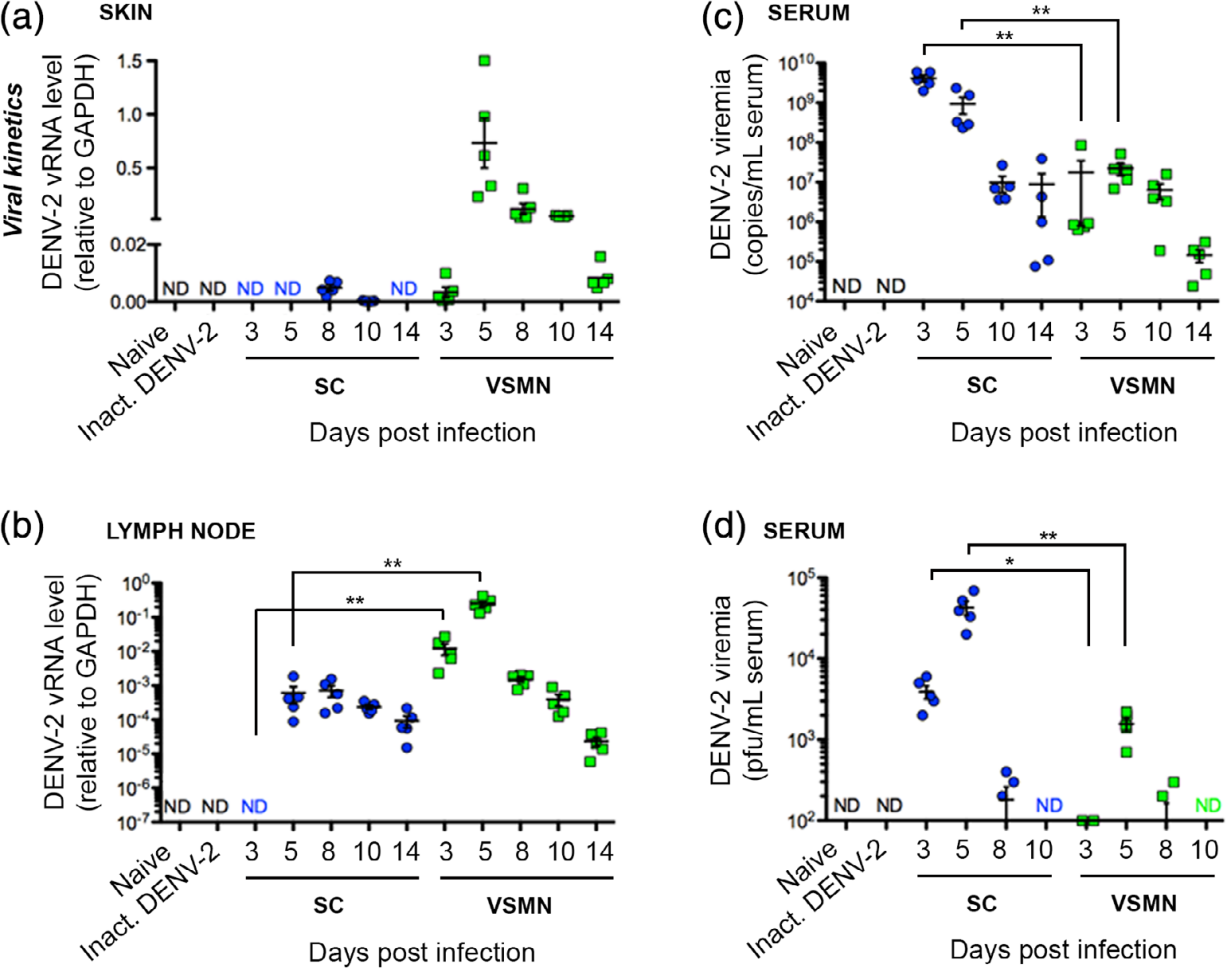
Intradermal delivery of DENV‐2 results in lower viremia but high tissue‐localized viral load and persistence relative to subcutaneous administration. DENV‐2 viremia kinetics for virus‐stabilized microneedle arrays (VSMN) intradermal and subcutaneous (SC) routes of delivery were evaluated by quantitative real‐time RT‐PCR of (a) ear skin, (b) draining lymph nodes, (c) serum, and (d) by plaque assay from serum. *n* = 5 AG129 mice per time point; ND, not detected; Mann–Whitney test, *p < 0.05, **p < 0.01

Following VSMN application to ventral ear skin for 15 min, mild erythema was observed in a few cases that faded within 48 hr; otherwise animals displayed no adverse reactions to MN penetration. DENV‐2 was shown to replicate in the skin, where peak viral RNA levels were detected at Day 5 post‐inoculation following VSMN delivery (Figure 3a). In contrast, viral RNA was below or at the lower limit of detection in the skin of animals inoculated subcutaneously. Likewise in the draining lymph nodes, viral load on Days 3 and 5 were significantly higher in animals inoculated via VSMN relative to subcutaneous controls (Figure 3b). Increased viral replication in draining lymph nodes following VSMN application may be attributed to the infection of dermal dendritic and epidermal Langerhans cells in the skin, which actively traffic to lymph nodes upon antigenic activation. These cells propagate virus while simultaneously priming the antiviral immune response through antigen presentation to adaptive immune cells. Viral persistence in lymph nodes was shown until the end of the assessed period at Day 14 for both administration routes (Figure 3b).

In contrast to localized virus detected at the site of inoculation as well as draining lymph nodes, viremia levels in the serum were significantly higher in animals inoculated subcutaneously relative to VSMN administration (Figure 3c and d). Virus administered subcutaneously showed peak systemic viral copy numbers of >10^9^ vRNA copies/mL of serum at Day 3 of infection, whereas virus administered intradermally by VSMN did not reach peak levels in the blood until Day 5 of infection, at 5 × 10^7^ vRNA copies/mL of serum. Collectively, these findings suggest that intradermal administration using VSMNs can be used to influence viral replication and localization and should be considered as an alternative to traditional administration routes. In fact, LAVs that produce lower systemic viremia are preferred for clinical safety. As such, VSMN‐mediated infection observed here that is low in the serum yet persists in the skin and lymph nodes would be desirable for a live‐attenuated dengue vaccine. In clinical patients, low persistent viremia following immunization with the yellow fever 17D vaccine has been shown to correlate with more robust virus‐neutralizing antibody recall responses.^66^ It was proposed that this was mediated by extended antigen availability in lymph nodes, which in the context of immunization, has been demonstrated to improve the affinity of antibody responses through strengthened germinal center reactions.^67^, ^68^

**Figure 4:**
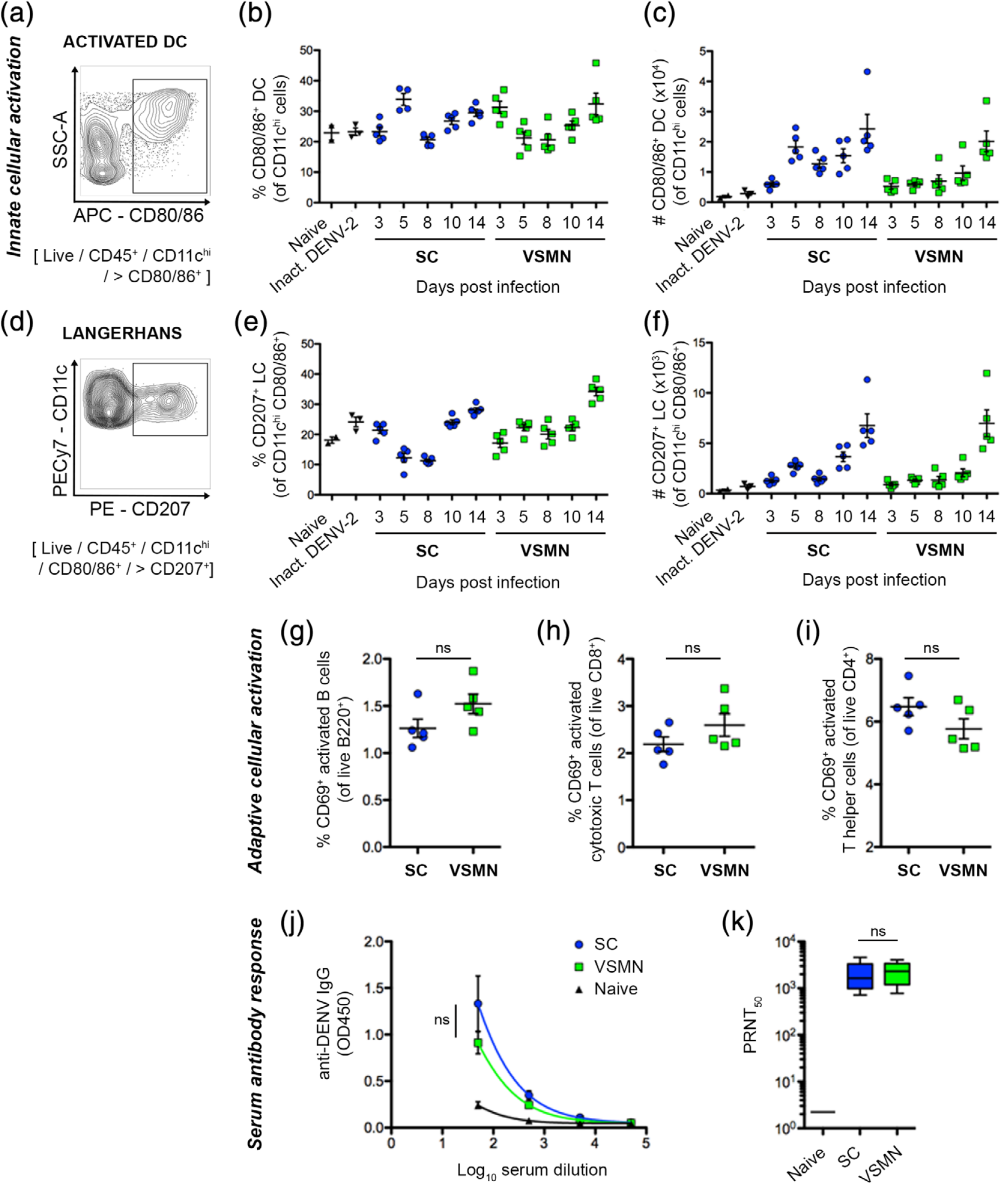
Virus‐stabilized microneedle array (VSMN) delivery of dengue virus (DENV) induces equivalent immune cell activation and serum antibody responses to subcutaneous administration. Innate cellular activation in draining lymph nodes was assessed by flow cytometric analysis of (a–c) activated dendritic cells, defined by CD80 and CD86 expression and presented as (a) representative plot, (b) proportion, and (c) number; and (d–f) activated Langerhans cells, defined by CD207 expression and presented as (d) representative plot, (e) proportion, and (f) number. Adaptive cellular activation in draining lymph nodes at Day 10 post‐DENV inoculation were assessed by flow cytometric analysis of activated (g) B cells, (h) CD8^+^ T cells, and (i) CD4^+^ T cells, as defined by CD69 expression; presented as proportion positive; *n* = 5 AG129 mice; ns, not significant. Serum antibody responses were assessed at Day 14 post‐DENV inoculation, as measured by (j) enzyme‐linked immunosorbent assay for total virus‐specific IgG antibody titer; and (k) plaque reduction neutralization assay (PRNT) for virus neutralization; data presented as box plots; *n* = 5 AG129 mice; ns, not significant

Innate and adaptive immune cell populations in draining lymph nodes were monitored in parallel following the different routes of infection. The proportion and number of activated dendritic cells (Figure 4a–c) and skin‐draining Langerhans cells (Figure 4d–f) followed broadly similar trends between administration routes over the course of infection. Similarly, no considerable differences were observed between VSMN and subcutaneous infection groups at Day 10 for activated B cell or CD4^+^ and CD8^+^ T cell populations, defined by CD69 up‐regulation (Figure 4g–i). Comparable cellular responses observed here indicate the noninferiority of VSMN to the benchmark standard of freshly prepared virus.

Robust humoral immunity, delineated by serum antibodies, is essential for cross‐serotype‐protection following dengue vaccination.^69^, ^70^ Serum analyses in this study indicated that dry stabilized virus delivered via VSMN elicited comparable antibody responses to freshly prepared virus delivered subcutaneously. No difference in either total anti‐dengue IgG antibodies (Figure 4j) or neutralizing antibody titer (Figure 4k) was observed between groups. These data are consistent with other MN‐based vaccine platforms where intradermal delivery has been demonstrated to induce comparable or superior serum antibody responses relative to intramuscular^71^ or subcutaneous^72^ vaccine administration.

### 2.4 VSMN‐mediated immunity protects from viral challenge

**Figure 5:**
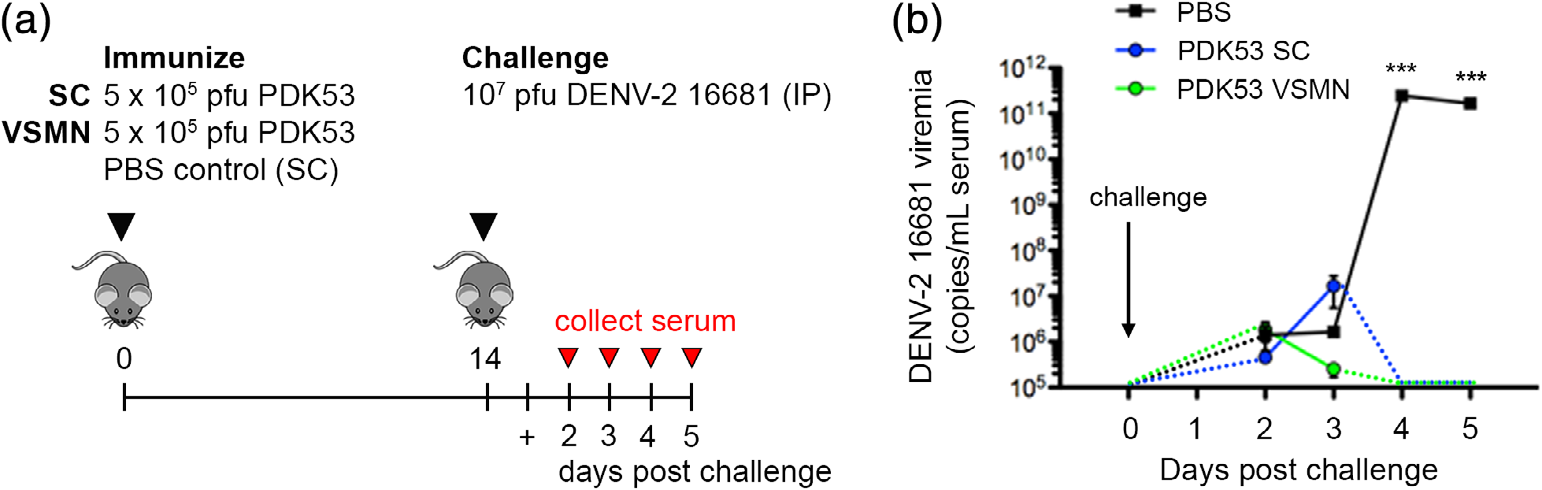
Virus‐stabilized microneedle array (VSMN)‐mediated vaccination mediates protection from viral challenge. (a) Protection from challenge was modeled using priming dose of DENV‐2 PDK53 administered via VSMN intradermal or subcutaneous (SC) routes, followed by intraperitoneal (IP) challenge with homotypic DENV‐2 16681. (b) Viremia measured by quantitative real‐time RT‐PCR of DENV‐2 vRNA in serum at Days 2–5 post‐challenge; *n* = 8 AG129 mice; ***p < 0.001, as analyzed by Kruskal–Wallis test of data on Days 4 and 5, respectively

An effective vaccine must provide protection from disease challenge. To demonstrate the quality of immunity generated by VSMN, we immunized animals with the DENV‐2 attenuated vaccine strain PDK53 by either VSMN or subcutaneous administration. Subcutaneous injection of PBS served as a negative unimmunized control. After 2 weeks, all groups were challenged with a high dose (10^7^ pfu) of the wild‐type DENV‐2 16681 strain by intraperitoneal inoculation and viremia was monitored in the serum for 5 days post‐challenge, as depicted (Figure 5a). Following challenge, control animals that received PBS during priming succumbed to rapid acute viremia exceeding 10^10^ vRNA copies/mL serum within 4 days (Figure 5b). In contrast, animals immunized with PDK53 by VSMN or by subcutaneous route were protected from disease, with complete viral clearance to below detectable levels by Day 4 postchallenge (Figure 5b). This confirms that immunization with stabilized live‐attenuated DENV delivered from our VSMN platform can achieve strong protective immunity that is noninferior to subcutaneous vaccination using fresh virus.

### 2.5 Towards long‐term ambient temperature storage

VSMNs provide the capacity for ambient temperature storage of dry formulated virus. In experiments conducted to validate VSMN‐mediated virus delivery and infection (Figures 2, 3, 4, 5), an optimized basic formulation of 0.5% CMC and 7.5% trehalose was used, which was demonstrated to support DENV‐2 viability over a 5‐day storage period (Figure 1f). Having established feasibility *in vivo*, we sought to further improve the ambient stability of DENV VSMN for extended storage by screening of additional excipients.

**Figure 6:**
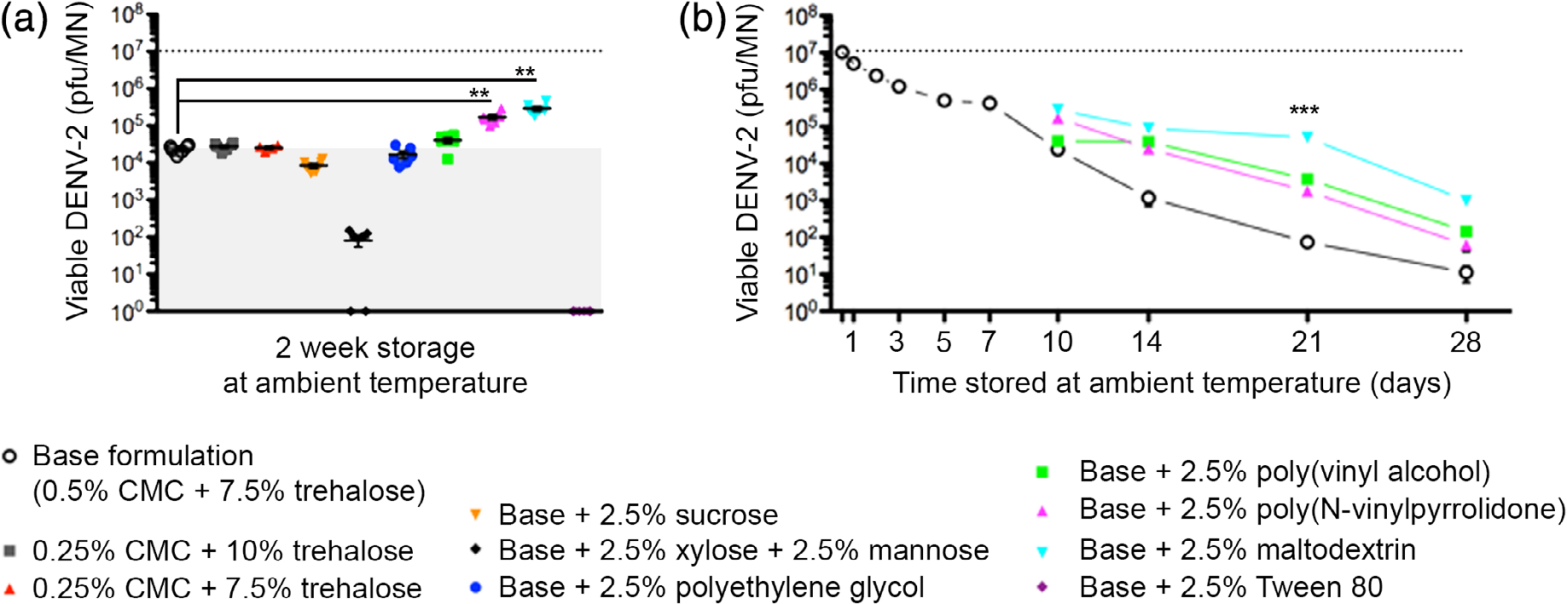
Dengue virus‐stabilized microneedle array (VSMN) formulation can be optimized for extended stability under ambient storage. DENV‐2 VSMN stability measured by plaque assay of solubilized virus following ambient temperature storage (25 ± 2 °C under vacuum with desiccant) for (a) 2 weeks; *n* = 6 VSMN per formulation; **p < 0.01, as analyzed by Mann–Whitney test, and (b) 1 month; *n* = 6 VSMN per condition; ***p < 0.001, as analyzed by Kruskal–Wallis test at Day 21. Dotted lines represent titer of viable DENV‐2 prior to storage; shaded area in (a) represents benchmark of base formulation

Excipients known to influence membrane stability, cryo‐ or thermo‐protection were added to the base formulation, then VSMN were produced as previously described with a single layer of stabilized DENV (Figure 6). First, VSMNs were stored at ambient temperature for 2 weeks then virus was solubilized from the arrays and viability was assessed by plaque assay. The inclusion of monosaccharides xylose and mannose, and the nonionic surfactant Tween 80 were shown to have deleterious effects on DENV‐2 viability relative to the base formulation. Conversely, the addition of poly(vinylpyrrolidone) (PVPON) or maltodextrin were found to provide further improvements to VSMN stability (Figure 6a).

Then, during extended storage conditions, the best performing VSMN formulation of 0.5% CMC, 7.5% trehalose and 2.5% maltodexrin was shown to preserve the viability of DENV‐2 for 3 weeks at ambient temperature, maintaining an infectious viral load of 10^5^ pfu/MN (Figure 6b). Maltodexrin is extensively used in the food industry and in the production of tableted pharmaceuticals as a heat‐tolerant stabilizer. In combination, maltodexrin and trehalose have previously been shown to aid the thermal stabilization of inactivated polio vaccine encapsulated in PLGA‐based microspheres,^73^ and trehalose is known to immobilize water molecules that protect viral protein structures during drying.^74^, ^75^, ^76^ We propose that the polysaccharides (CMC, trehalose and maltodextrin) and water‐soluble synthetic polymers (poly(vinyl alcohol) [PVA] and PVPON) used in this study protect DENV structure from complete dehydration through their ability to retain structured water molecules in the matrix. The hydroxyl groups of the sugars interact with viral proteins and polysaccharides, thereby mimicking water‐protein and water‐polysaccharide interactions of virus in solution.^75^, ^76^

### 2.6 Conclusions

The stability of virus particles to retain infectivity is critical for LAV efficacy and the protection of immunized patients. Stabilizing excipients for MN‐based platforms described here will be crucial to the design of successful live virus formulations. This work demonstrates that inherently unstable viruses such as DENV can be stabilized for ambient temperature storage in a compact, dry film form using polymeric formulations that can be directly and effectively applied to the skin with a syringe needle‐free patch. Using tailored saccharide formulations and well‐known excipients already utilized for human applications, DENV viability at ambient temperature was successfully improved from hours to weeks, and was shown to elicit strong protective immunity following intradermal administration of stabilized form. VSMN strategies can be broadly applied to other viruses and should serve as a tool to improve LAVs destined for CTC immunization campaigns, including next‐generation vaccines against dengue and other mosquito‐borne diseases. Ultimately, technologies such as the one reported here have the potential to extend immunization coverage by virtue of better vaccine usability and cold‐chain independence.

## 3 MATERIALS AND METHODS

### 3.1 Mice

Animal studies were conducted at the National University of Singapore in accordance with Institutional Animal Care and Use Committee regulations and approvals. AG129 (Interferon type I/II receptor‐deficient) mice breeders were purchased from B&K Universal and bred at InVivos (Singapore) under specific pathogen‐free conditions; 7‐ to 10‐week‐old female AG129 mice were used for experiments.

### 3.2 Viruses

DENV strains DENV‐2 16681 (U87411.1), DENV‐2 PDK53 (M84728.1), DENV‐4 2270 and Yellow Fever vaccine strain YF‐17D (X03700.1) were used. Viruses were propagated in *Aedes albopictus* C6/36 cells in 2% fetal bovine serum (FBS) until cytopathic features were observed, followed by membrane filtration concentration (Vivacell 100, Sartorius, Goettingen, Germany).

### 3.3 Polymers and polysaccharides

Saccharide formulations included the following: CMC sodium salt (medium viscosity, meets United States Pharmacopeial Convention (USP) testing specification), d‐[+]‐Trehalose dihydrate (from *Saccharomyces cerevisiae*, ≥99%), Lutrol F68 (Kolliphor P 188), d‐(+)‐Xylose (BioUltra, ≥99%, sum of enantiomers, by high‐performance liquid chromatography), d‐(+)‐Mannose (BioUltra, ≥99.5%, sum of enantiomers, by high‐performance liquid chromatography), polyethylene glycol (*M*_n_ = 3,350 Da), PVA (*M*_w_ = 89–98 kDa, 99% hydrolyzed), PVPON (average *M*_w_ = 40 kDa), maltodexrin, all sourced from Sigma‐Aldrich (St Louis, MO, United States); Sucrose (d‐(+)‐Saccharose, AR grade) (Fisher Chemical, United Kingdom); and Tween 80 (AppliChem GmbH, Darmstadt, Germany).

### 3.4 Fabrication and storage of VSMN

Polydimethylsiloxane molds (Sylgard 184, Dow‐Corning) were generated by laser micromachining,^77^ encompassing an array of 77 conical MNs (250 μm base, 750 μm height and 600 μm pitch) arranged in alternating rows of 9 and 8. Arrays were fabricated by melting poly‐l‐lactide (IV 1.8 dlg^−1^, Polysciences Inc., Warrington, PA, United States) over molds under vacuum (−20 in. Hg, 200 °C, 40 min), followed by rapid hardening (−20 °C, 30 min), then equilibration to room temperature. Arrays were plasma treated (air plasma, 60 s) prior to virus deposition. Concentrated virus stock was mixed with saccharide formulations at 1:1 volume ratio, then 100 μL was pipetted onto the array surface and dried until completion at 25 °C. This process was repeated for additional layers as indicated. Unformulated control MN comprised virus stock mixed with Roswell Park Memorial Institute medium (RPMI). Dry VSMN were stored at −80 °C or as described for up to 4 weeks. Ambient temperature was defined as conditions on the laboratory bench (stored in a desiccator under vacuum; temperatures monitored to fluctuate between 23 and 28 °C). For storage studies, VSMN were fabricated and stored for time periods indicated, then transferred to −80 °C for the remainder of the experiment.

### 3.5 Skin application of VSMN

Animals were anesthetized and VSMN were applied to the right ear by pressing down vertically with the thumb and index finger, ensuring even penetration of the array to the flat area of the ventral ear skin for 15 min, then removed. Control animals received a matched viral dose of live or inactivated DENV in 200 μL endotoxin‐free phosphate‐buffered saline (PBS), administered subcutaneously to the scruff. For all groups, the draining lymph nodes collected for analyses were the right side axillary and brachial lymph nodes.

### 3.6 Confocal microscopy

Microscopy was performed using a Zeiss Axio Observer Z1 LSM 700 (488 nm laser) at 10× objective. Normalized signal intensities were determined by integrating the total confocal fluorescence signal from z‐stacks collected through the length of individual MNs and normalizing to the total fluorescence of MNs prior to skin application. Image processing and data analysis was performed using Image J.

### 3.7 Plaque assay

Infectious viral titer was assessed for propagated virus stocks, VSMN load, and mouse serum samples following infection. Virus was released from VSMN by solubilizing deposited material in 500 μL RPMI + 3% FBS (37 °C, 30 min, gentle agitation) in a 24‐well plate. For plaque assays, serial log dilutions of samples were performed in RPMI with 3% FBS. Baby hamster kidney fibroblast BHK‐21 cells (American Type Culture Collection) grown to 95% confluency in 24‐well plates were then infected with 200 μL of diluted virus and incubated for 2 hr at 37 °C with 5% CO_2_. Supernatant was removed, then cells were layered with 1 mL prewarmed RPMI with 2% CMC (Sigma‐Aldrich) supplemented with 1% pen/strep (100×, Gibco, Thermo Fisher Scientific, Waltham, MA, United States) and incubated for 5 days at 37 °C with 5% CO_2_. Following incubation, cells were fixed and stained with 10% formaldehyde solution with 0.5% crystal violet for 1 hr at room temperature, then washed under running water and dried. Plaques were counted and pfu per mL or per MN were calculated.

### 3.8 Quantitative real‐time RT‐PCR

Viral RNA (vRNA) was isolated from serum using the QIAamp Viral RNA Mini kit (Qiagen, Hilden, Germany). RNA from tissues was extracted using the RNeasy Mini Kit (Qiagen). Quantification of viral genomes or host transcripts was performed using the iTaq Universal SYBR Green One‐Step Kit (Bio‐Rad, Hercules, CA, United States). Viral titers from serum, expressed as copies per mL, were calculated in relation to DENV‐2 plasmid standards (5′‐ACACCACAGAGTTCCATCACAGAAGCAGAACTAACAGGCTATGGCACTGTCACGATGGAATGCTCTCCGAGAACGGGCCTCGACTTCAATGAGATG‐3′). Viral titers from tissues were expressed relative to host transcript GAPDH. Primer sequences were: DENV‐2 envelope (GAAGCCAAACAGCCTGCC/GGAGTGTTTGCAGACGAACC); mGAPDH (TTGATGGCAACAATCTCCAC/CGTCCCGTAGACAAAATGGT).

### 3.9 Flow cytometry

Spleen and lymph node single cell suspensions were stained for viability discrimination in PBS for 15 min (Live/Dead Near‐IR Dead Cell Stain Kit, Thermo Fisher Scientific), then resuspended in staining buffer (PBS, 0.5% bovine serum albumin, 0.05% sodium azide) at ~10^6^ cells/100 μL. Fc receptors were blocked with purified anti‐mouse CD16/32 for 15 min, then cells were stained with directly‐conjugated antibody cocktails for 1 hr at 4 °C using the following panels. Dendritic cell activation: CD45‐PerCPCy5.5 (104), CD11c‐PECy7 (N418), I‐A/I‐E‐AlexaFlour488 (M5/114.15.2), CD80‐APC (16‐10A1), CD86‐APC (GL‐1), CD207‐PE (4C7). T and B cell activation: CD4‐APC (GK1.5), CD8a‐FITC (53–6.7), B220‐PECy7 (RA3‐6B2), CD69‐PerCPCy5.5 (H1.2F3). All antibodies were sourced from Biolegend (San Diego, CA, United States). Data were acquired on a BD LSR II flow cytometer (BD Biosciences, San Jose, CA, United States) and frequencies of activated cell populations were determined using FlowJo analysis software (FlowJo LLC, Ashland, OR, United States). Gating for immune cell populations are outlined in Supporting Information Figures S1 and S2.

### 3.10 Plaque reduction neutralization assay

DENV neutralizing antibody titers of mouse serum were determined by plaque reduction neutralization assay (PRNT). Briefly, serial dilutions of heat‐inactivated serum (56 °C, 20 min) were performed in RPMI with 1% FBS, prior to incubation with 50 pfu DENV‐2 16681 for 2 hr at 37 °C. BHK‐21 cell monolayers were infected with 200 μL incubated virus for 1 hr at 37 °C. Cells were overlaid with RPMI/2% CMC/1% pen/strep, incubated for 5 days, then plaques were stained and counted as described for the plaque assay. The serum dilution resulting in 50% inhibition of plaque formation relative to virus only control (PRNT_50_) was determined in Prism using a nonlinear regression with variable slope (4 parameters) constrained to 100 and 0 for top and bottom, respectively.

### 3.11 Viral challenge

Mice were immunized with 5 × 10^5^ pfu DENV‐2 PDK‐53 (attenuated vaccine strain) via VSMN intradermal or subcutaneous administration, as described above. Control animals received subcutaneous administration of 200 μL endotoxin‐free PBS. All groups were challenged on Day 14 with 1 × 10^7^ pfu DENV‐2 16681 (parental wild‐type strain) by intraperitoneal injection. Serum viremia was analyzed as a measure of protection up to 5 days post‐challenge by quantitative PCR.

### 3.12 Statistical analysis

Data sets were analyzed using *t* tests, Mann–Whitney tests or Kruskal–Wallis tests using Prism (GraphPad Software, San Diego, CA). Unless otherwise indicated, data is presented as mean ± *SEM*. with statistical significance defined by *p*‐values; *p < 0.05, **p < 0.01, ***p < 0.001.

## CONFLICT OF INTERESTS

The authors have no conflicts of interest to declare.

## AUTHOR CONTRIBUTIONS

M.E.T., D.S.S.M.U., and P.T.H. designed the experiments, analyzed the data, and performed statistical analyses. M.E.T., D.S.S.M.U., and A.R.M.S. fabricated MNs. D.S.S.M.U. designed and prepared polymer formulations. D.S.S.M.U. and A.R.M.S. carried out microscopy. M.E.T. performed stability assays, in vivo experiments and analyses (viral kinetics, serum responses and flow cytometry). K.B., S.A., and E.E.O contributed to design and interpretation of viral kinetics studies in AG129 mice. M.E.T. and P.T.H. wrote the manuscript.

## Supporting information


**Figure S1** Immunophenotyping gating strategy for dendritic and Langerhans cells isolated from skin draining lymph nodes

**Figure S2** Immunophenotyping gating strategy for activated T and B populations isolated from lymph nodes and spleen
