# Supplementary figures and images for: Microneedle‐based intradermal delivery of stabilized dengue virus
Source: Bioeng Transl Med. 2019 Feb 25;4(2):e10127. doi: 10.1002/btm2.10127 (PMC6584444; doi:10.1002/btm2.10127)

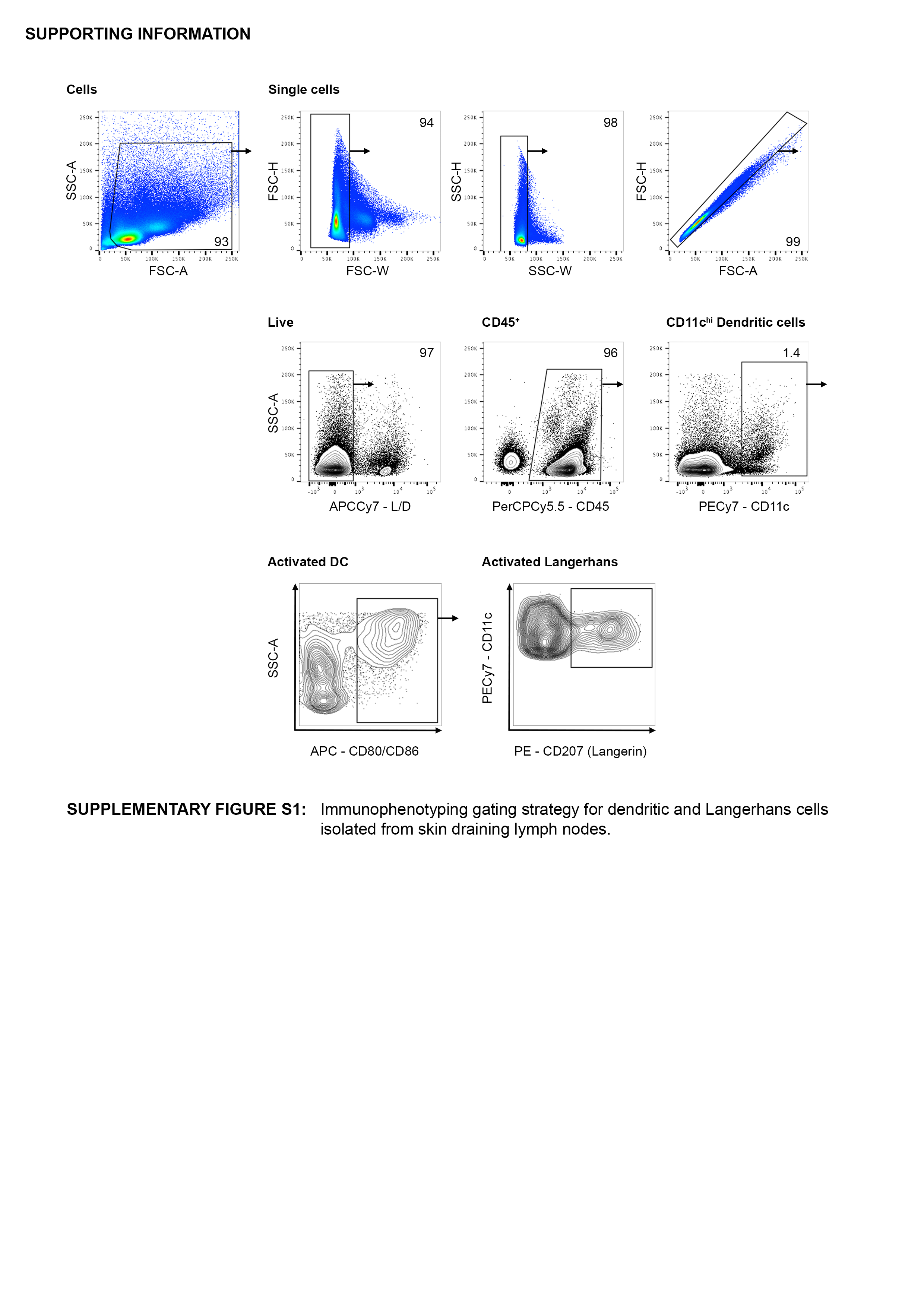

Supplement: Supplementary file 1 — Figure S1 Immunophenotyping gating strategy for dendritic and Langerhans cells isolated from skin draining lymph nodes [file BTM2-4-na-s001.tif]

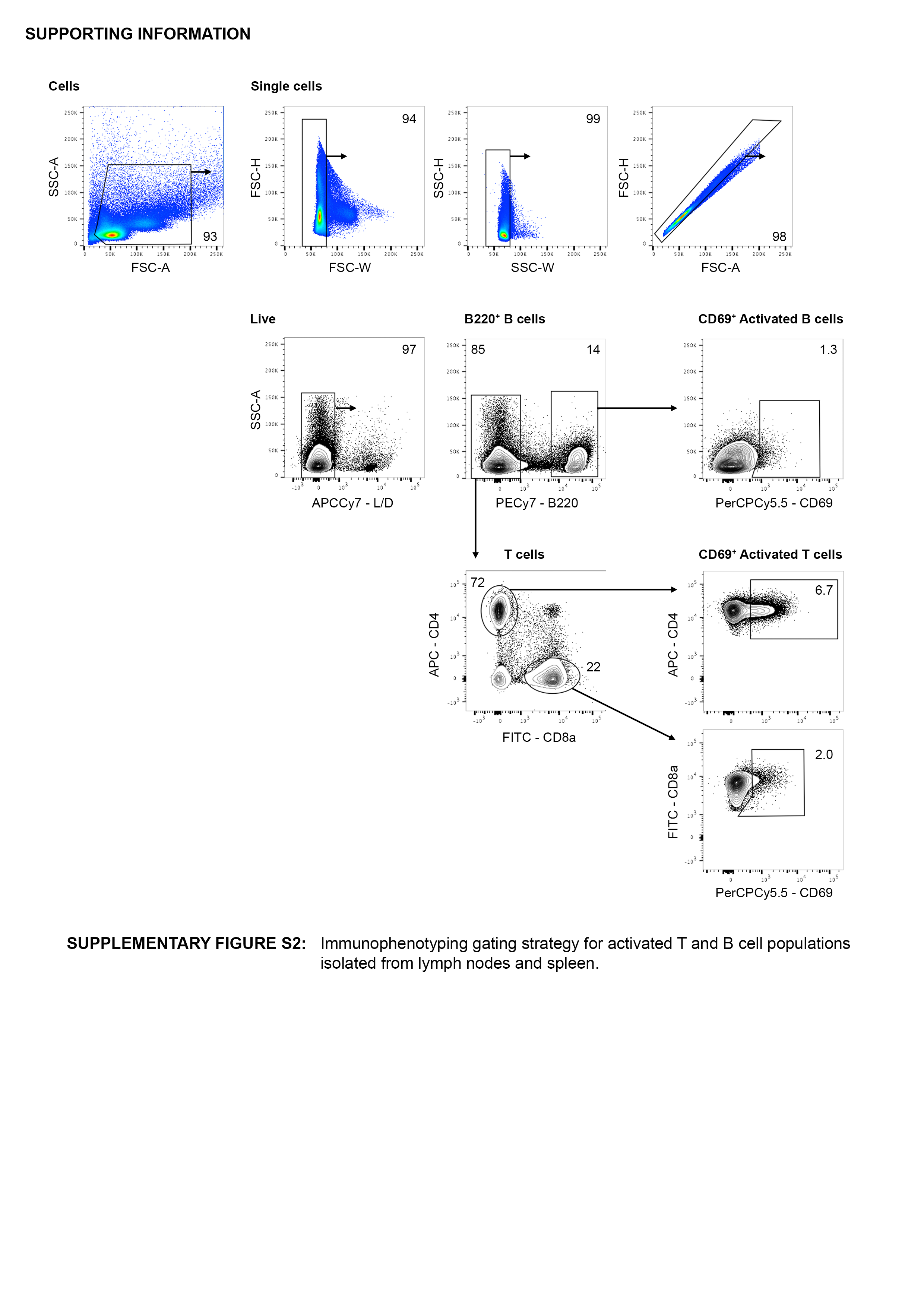

Supplement: Supplementary file 2 — Figure S2 Immunophenotyping gating strategy for activated T and B populations isolated from lymph nodes and spleen [file BTM2-4-na-s002.tif]
